# Molecular Fingerprinting for Source Attribution of Nanoplastics in Drinking-Water Systems

**DOI:** 10.3390/molecules31152610

**Published:** 2026-07-27

**Authors:** José Roberto Vega-Baudrit, Mary Lopretti, Felipe Orozco

**Affiliations:** 1 National Nanotechnology Laboratory (LANOTEC), National Center for High Technology (CeNAT), CONARE, San José 10109, Costa Rica; felipeorozcogutierrez@gmail.com; 2 Escuela de Química, Universidad Nacional, Heredia 86-3000, Costa Rica; 3 Laboratory of Nuclear Techniques Applied to Biochemistry and Biotechnology, Nuclear Research Center, Faculty of Sciences, University of the Republic (UdelaR), Mataojo 2055, Montevideo CP 11400, Uruguay; mlopretti@gmail.com

**Keywords:** nanoplastics, drinking water, source attribution, molecular fingerprinting, polymer aging, packaging, water treatment, QA/QC, analytical chemistry, chemometrics

## Abstract

Detection of nanoplastics in drinking-water systems is only the first analytical step toward exposure interpretation; the next challenge is source attribution. This review examines molecular fingerprinting and transformation pathways that can link nanoscale polymer signals to source waters, drinking-water treatment, distribution infrastructure, packaging materials, laboratory background, or aging processes across the potable-water chain. Nanoplastics are treated here as operationally defined particles below 1 µm, including intentionally manufactured primary nanoplastics and secondary nanoplastics generated by fragmentation, abrasion, weathering, treatment, storage, or packaging stress. The synthesis evaluates how polymer identity, particle morphology, surface oxidation, additive and oligomer profiles, thermal degradation markers, matrix context, and quality assurance/quality control (QA/QC) can be combined into defensible source assignments. Analytical platforms considered include surface-enhanced Raman spectroscopy (SERS), atomic force microscopy–infrared spectroscopy (AFM-IR), optical photothermal infrared spectroscopy (O-PTIR), stimulated Raman scattering microscopy (SRS), pyrolysis–gas chromatography–mass spectrometry (Py-GC/MS), asymmetric flow field-flow fractionation coupled to Py-GC/MS (AF4-Py-GC/MS), matrix-assisted laser desorption/ionization time-of-flight mass spectrometry (MALDI-TOF-MS), and chemometric workflows. The central conclusion is that source attribution cannot be inferred from polymer identity alone; robust interpretation requires convergent evidence from particle-level chemistry, polymer-specific mass, additive or marker-ion signatures, aging state, blanks, recovery, and contextual sampling design.

## 1. Introduction: From Analytical Detection to Source Attribution

Nanoplastics in drinking water are no longer merely a question of detectability. In this review, nanoplastics are defined operationally as polymer-containing particles below 1 µm, recognizing that practical lower and upper size boundaries remain method-dependent. Primary nanoplastics are intentionally produced at the nanoscale, whereas secondary nanoplastics arise from fragmentation, abrasion, weathering, treatment-induced transformation, packaging stress, or sample-handling processes. Recent analytical advances have shown that polymeric particles and polymer-like nanoscale signals can be detected in bottled, tap, and other potable water, as well as in potable water treatment systems and environmentally relevant aqueous matrices [1,2,3,4,5,6,7,8,9,10,11,12,13]. Yet detection alone is scientifically incomplete. A drinking-water result is not fully interpretable until the observed particle population can be connected to a plausible source or transformation pathway. Without source attribution, numerical particle counts or polymer-specific mass concentrations remain difficult to translate into control strategies, monitoring design, or exposure reduction.

This review, therefore, moves from the question “Can nanoplastics be measured?” to the more demanding question “What does the measured signal mean?” The distinction is not semantic. A poly(ethylene terephthalate) (PET) signal in bottled water may indicate bottle-wall abrasion, cap or liner contribution, filtration materials, source-water contamination, or analytical background. Polyethylene (PE), polypropylene (PP), polystyrene (PS), polyamide (PA), poly(vinyl chloride) (PVC), PET, and recycled PET (rPET) signals may reflect environmental input, treatment-train transformation, membrane or pipe materials, packaging components, airborne particles, or contamination introduced during sampling. A nanoscale particle with a Raman or SERS spectrum similar to a reference polymer may have undergone oxidation, biofilm coating, additive loss, or fragmentation that changes both its surface chemistry and its source relevance [4,7,8,9,10,11,12,13,14,15].

Source attribution is, therefore, a chemical forensic problem. It requires a chain of evidence that links polymer identity, morphology, size distribution, surface functionality, additive or oligomer markers, thermal decomposition products, matrix characteristics, and QA/QC controls. Polymer identification is necessary but not sufficient. The same base polymer can enter drinking water through several pathways, while aging and treatment can modify the diagnostic signatures that analysts use to infer origin [16,17,18,19,20,21,22].

Existing reviews have substantially advanced the field by summarizing occurrence, toxicological concern, sampling limitations, spectroscopy, thermal analysis, and the broader metrology of micro- and nanoplastics. However, most reviews still treat source interpretation as a downstream implication rather than as an explicit analytical task. The gap addressed here is the conversion of a confirmed nanoplastic signal into a defensible source hypothesis. This requires distinguishing occurrence, polymer identification, process-induced transformation, and source attribution as separate evidentiary levels rather than treating them as one result.

This article is written as a critical, narrative synthesis rather than as a formal systematic review. Its scope is deliberately restricted to the source-attribution step: molecular transformation, chemical fingerprinting, packaging-derived particles, treatment-induced alteration, QA/QC controls, and data-fusion strategies. The article is organized into ten sections, including source mapping, aging mechanisms, molecular fingerprints, source-apportionment strategies, packaging contributions, treatment effects, monitoring implications, a research agenda, and conclusions.

Review scope and literature-selection approach. The literature was selected to prioritize studies that directly inform drinking-water nanoplastics, source tracing, polymer aging, packaging release, treatment-induced transformation, molecular fingerprinting, QA/QC, and method comparability. Primary drinking-water and bottled-water studies were combined with transferable methodological studies in aqueous matrices, polymer-aging studies, and recent work on reference materials and data-driven classification. This approach was not designed to generate pooled occurrence estimates; it was designed to build an evidence framework for source attribution, where matrix context, polymer identity, aging state, and analytical controls must be interpreted together.

## 2. Sources of Nanoplastics in Drinking-Water Systems

Drinking-water nanoplastics can originate before, during, or after treatment. Source waters may carry secondary fragments generated by weathering, abrasion, wastewater effluent, agricultural runoff, atmospheric deposition, and the ongoing fragmentation of legacy plastic debris [1,2,3]. The polymer classes most relevant to the present source-mapping framework include PET/rPET, PE, PP, PS, PVC, PA, polyester fibers, coatings, pigments, cap-liner materials, membrane-associated polymers, and aged or biofilm-coated particles [7,8,9,10,11,12,13,14,15,16]. Treatment plants may remove a fraction of that input, but they may also alter particle size, surface chemistry, aggregation state, and detectability. Distribution systems add another layer of complexity because pipes, deposits, seals, valves, storage tanks, and biofilms can influence particle transport and retention. Bottling and consumer handling can introduce packaging-derived particles even after water has met conventional quality criteria [7,8,9,10,11,12,13,14,15].

Attribution becomes difficult because source pathways can converge into the same operational nanoscale fraction. A particle isolated below 1 µm may be a secondary fragment from aquatic debris, a stress-generated packaging fragment, a treatment-aged particle, a natural colloid with adsorbed polymeric material, or a laboratory contaminant. For this reason, source categories should be treated as hypotheses rather than as labels. The evidence needed to support each hypothesis differs. Packaging attribution requires information on polymer compatibility with bottle, cap, liner, or filtration components; treatment attribution requires sampling before and after unit operations; distribution attribution requires spatial sampling and infrastructure metadata; and source-water attribution requires upstream controls and time-resolved sampling [10,11,12,13,14,15].

The strongest evidence currently comes from studies that connect polymer identity with matrix context. PET nanoplastics identified in commercially bottled drinking water by SERS support a packaging-related hypothesis, but even then, the exact stage of generation cannot be determined automatically [7]. SRS imaging of bottled water substantially increased the detectable particle population and demonstrated the value of rapid single-particle chemical imaging, but it also reinforced that analytical selectivity and library coverage determine which particles become source-informative [8].

In treatment plants, AFM-IR and Py-GC/MS have provided complementary particle- and mass-level evidence for PE and PVC nanoplastics, suggesting that unit processes may function not only as barriers but also as transformation zones [10,13].

**Figure 1:**
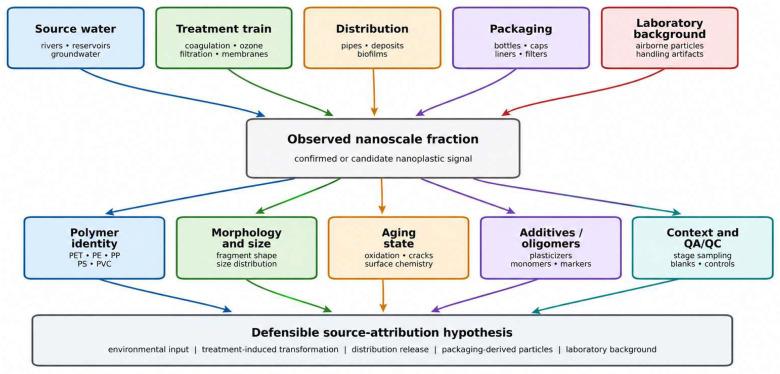
Source-attribution map for drinking-water nanoplastics. The observed nanoscale fraction can integrate particles from source water, treatment operations, distribution infrastructure, packaging, and laboratory background. Defensible source attribution requires convergent evidence from polymer, condition, additive, and contextual sources rather than polymer identity alone.

Figure 1 summarizes this attribution problem as a convergent chain. The observed nanoscale fraction is not a direct mirror of a single source. It is a composite analytical object resulting from environmental inputs, treatment, distribution, packaging, and laboratory handling. A source claim becomes credible only when polymer evidence is combined with condition, additive, contextual, and QA/QC controls.

Table 1 formalizes this logic by separating candidate sources, expected material signals, source-informative evidence, and the main ambiguity associated with each attribution hypothesis. It also clarifies that source categories should be evaluated as testable hypotheses rather than as labels attached to a single polymer match. The table is intended to prevent overinterpretation of polymer identity by showing why origin claims require contextual, material, and QA/QC evidence in addition to chemical confirmation.

### Specific Challenges of Nanoscale Source Attribution Compared with Microscale Plastics

Source attribution at the nanoscale is not a simple extension of microplastic source tracing. Microscale particles often preserve visible morphology, color, fragment shape, and sometimes use-related wear patterns; nanoplastics provide far less mass per particle, weaker spectral signal, higher susceptibility to aggregation, and greater losses through adsorption to glassware, filters, and tubing. Natural colloids, dissolved organic matter, biofilms, and treatment residuals can also mask or modify nanoscale polymer signatures. Therefore, nanoplastic source attribution depends more strongly on controlled preprocessing, blanks, recovery, matrix-matched validation, and convergent chemical evidence than typical microscale particle attribution.

The framework developed for drinking-water systems can be transferred to other relatively clean aqueous matrices such as bottled beverages, groundwater, surface water, or treated wastewater, but not without adaptation. Matrices with higher organic matter, salts, biological material, or suspended solids require stronger separation, digestion, and blank-correction strategies. Thus, the framework should be interpreted as a portable evidence logic rather than as a universal protocol.

## 3. Polymer Aging and Transformation Mechanisms

Source attribution must account for the chemical dynamics of polymer particles. Aging can occur in source waters, during treatment, within distribution systems, during storage, and even during sample preparation. Photo-oxidation, chlorination, ozonation, UV-based advanced oxidation, mechanical abrasion, thermal stress, and biofilm formation can modify particle surfaces, alter aggregation behavior, and change the analytical response measured by spectroscopy or mass spectrometry [17,18,19,20,21,22,25]. For attribution purposes, aging should therefore be interpreted as a process history rather than as a generic degradation label.

Polymer aging is polymer-specific. Polyolefins such as PE and PP, as well as PS, commonly develop oxygen-containing functional groups under photo-oxidative or oxidative conditions, including carbonyl, hydroxyl, and carboxyl-related signatures. PET and rPET may instead show a combination of surface oxidation, hydrolysis-related changes, chain scission, and changes in oligomer or additive release. PA and polyester fibers may also show amide- or ester-related changes that alter IR/Raman interpretation. Consequently, carbonyl indices, band shifts, and surface-chemistry markers must be interpreted in relation to polymer class, aging environment, and analytical method rather than used as universal indicators of origin [20,21,22]. Aged particles may also be retained differently by filters, react differently during digestion, or yield altered marker-ion patterns during pyrolysis [19,20,21,22].

Treatment chemistry is especially important for drinking-water systems. Ozonation and UV-based processes can oxidize polymer surfaces and may promote fragmentation or changes in hydrophilicity, whereas chlorination can react with polymer surfaces and with leached monomers, oligomers, plasticizers, stabilizers, or other organic additives. These reactions may modify the pool of potential disinfection by-product precursors, but they should not be interpreted as a simple conversion of monomers into oligomers or additives. Their magnitude and diagnostic value depend on polymer type, previous aging, disinfectant dose, pH, contact time, and matrix chemistry [17,18,19]. These processes change particle fate and can also strengthen or obscure the interpretation of the source.

**Figure 2:**
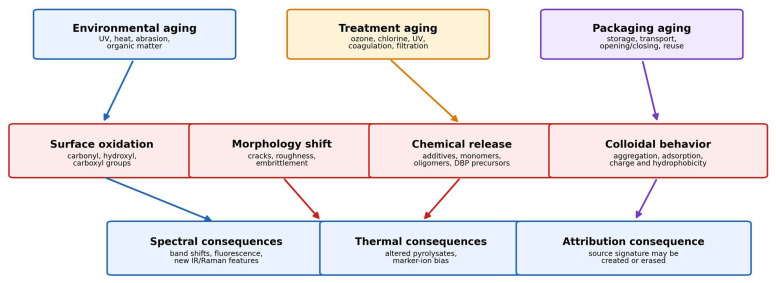
Chemical aging pathways and analytical consequences for nanoplastics in drinking-water systems. Environmental, treatment-related, and packaging-related aging can produce surface oxidation, morphological change, additive or oligomer release, and altered colloidal behavior. These processes affect spectral features, pyrolysis products, and source-attribution confidence.

Figure 2 summarizes the major transformation pathways and their analytical consequences. Aging can produce useful fingerprints, such as carbonyl formation, surface roughening, or additive loss, but it can also erase source signatures by making different polymers more similar in surface chemistry or by masking diagnostic features with matrix-derived coatings. The central analytical challenge is therefore not simply to detect aging, but to determine whether the observed aging state is source-informative, source-neutral, or source-obscuring.

Table 2 translates these mechanisms into a process-specific attribution framework. For each treatment or handling process, it distinguishes the expected transformation, the potential attribution value, and the analytical caution needed to avoid assigning a treatment origin when similar chemical changes could also arise from environmental aging, storage, or laboratory handling. The row-level references indicate that attribution should be process-tested rather than assumed from surface oxidation alone.

## 4. Molecular Fingerprints and Chemical Markers

Molecular fingerprinting converts a particle or polymer mass signal into source-relevant evidence. The fingerprint can be spectral, thermal, morphological, additive-based, or contextual. Spectral approaches include Raman microscopy, SERS, FTIR-related methods, AFM-IR, O-PTIR, and SRS; thermal and mass-based approaches include Py-GC/MS, AF4-Py-GC/MS, and MALDI-TOF-MS; morphological and size-resolved approaches include SEM, AFM, nanoparticle tracking analysis (NTA), dynamic light scattering (DLS), and fractionation workflows [4,22,23,24,25,26,27,28,29,30,31,32,33,34,35]. Spectral tools preserve particle-level identity but can suffer from fluorescence, weak nanoscale signal, and library mismatch. Thermal tools provide stronger polymer-mass evidence but destroy particle morphology. Morphological tools support size and shape interpretation but are insufficient without chemical confirmation.

The value of a fingerprint depends on specificity and independence. PET, rPET, PE, PP, PS, PVC, PA, polyester fibers, and pigmented or coated polymer fragments can often be distinguished by characteristic spectral or pyrolysis features, but aged materials, additives, pigments, biofilms, and matrix residues complicate matching. A source-attribution workflow should therefore not treat a library match as the endpoint. It should document spectral preprocessing, match thresholds, blank correction, polymer-marker selection, recovery, and the potential for aged or additive-rich particles to deviate from pristine reference materials [4,22,32].

Additives and oligomers are underused attribution evidence. Plasticizers, stabilizers, pigments, catalysts, dyes, processing aids, and oligomeric fragments can be more source-informative than the base polymer when multiple products share the same polymer class. For example, a PET-based signal alone does not distinguish the bottle wall from the filtration material; additive or oligomer patterns, particle morphology, and storage history may improve discrimination. Similarly, colored or coated fragments may implicate caps, labels, or closure materials when their pigment or coating signatures are compatible with the packaging component [15,16].

The most defensible fingerprinting strategy is tiered and cross-validated. First, establish that the signal is plastic rather than a natural colloid or laboratory contaminant. Second, assign polymer class using particle-level or mass-based chemistry. Third, evaluate aging state and morphology. Fourth, compare additive, oligomer, or pyrolysis-marker patterns against plausible source materials. Fifth, use a contextual sampling design to test whether the source hypothesis survives blanks, replicates, before/after gradients, and uncertainty assessment.

Table 3 maps major polymer and material classes to plausible drinking-water sources and fingerprint dimensions. It should be read as a decision aid rather than as a deterministic source key: the purpose is to identify which spectral, thermal, morphological, additive, or contextual evidence can strengthen or weaken a source hypothesis. The table also defines rPET and PA explicitly and distinguishes base polymers from pigmented, coated, aged, or biofilm-associated materials.

### Critical Comparison of Analytical Evidence Classes

For source attribution, analytical techniques should be compared by the type of evidence they generate rather than by sensitivity alone. Raman, SERS, AFM-IR, O-PTIR, and SRS can support particle-level chemical identity; Py-GC/MS, AF4-Py-GC/MS, and MALDI-TOF-MS can support polymer-specific mass, marker ions, and oligomer evidence; SEM, AFM, NTA, DLS, and fractionation can support morphology, size distribution, and colloidal behavior. None of these evidence classes is sufficient alone. A high-confidence source assignment requires convergence between chemical identity, process-compatible markers, particle or mass information, QA/QC controls, and sampling context [22,23,24,25,26,27,28,29,30,31,32,33,34,35,36,37,38,39,40,41].

## 5. Source-Apportionment Strategies

Source apportionment should be framed as a ranking of competing hypotheses. A single polymer match rarely provides enough evidence to claim origin. Instead, the analyst should ask which source hypothesis best explains the full set of evidence: polymer class, particle size, morphology, aging state, additive or marker-ion profile, matrix context, blanks, recovery, and spatial or process-stage gradients. This approach is closer to environmental forensics than to simple polymer identification.

A practical source-attribution workflow has six operational steps. Step 1 is exclusionary QA/QC: remove implausible interpretations by using field blanks, procedural blanks, laboratory air controls, non-plastic colloid checks, and polymer-compatible sampling materials. Step 2 is polymer confirmation: establish polymer identity and quantitative endpoint through independent analytical modalities, such as SERS plus AFM-IR, or particle imaging plus Py-GC/MS. Step 3 is aging-state assessment: evaluate oxidation, surface roughness, embrittlement, additive loss, or biofilm coating. Step 4 is source-material comparison: compare particles with bottle, cap, liner, pipe, membrane, or source-water materials. Step 5 is contextual gradient testing: evaluate before/after treatment, storage, opening, cap-rinse, bottle-rinse, and distribution gradients. Step 6 is confidence-level assignment using the evidence hierarchy in Table 4 [23,24,33,34,35,36,37,38,39,40,41].

Machine learning (ML) and chemometrics can help, but they do not remove the need for chemically meaningful features. Automated spectral classification is useful only when training libraries include aged polymers, additives, pigments, biofilm-associated particles, and matrix interferences. NTA and DLS can support size and aggregation screening but should not be interpreted as polymer-specific evidence without chemical confirmation. AI-assisted holographic microscopy, SRS imaging, Raman classification, and multimodal datasets are promising because they can increase throughput and standardize decision rules, but source attribution still requires transparent uncertainty reporting [24,36,37,38].

**Figure 3:**
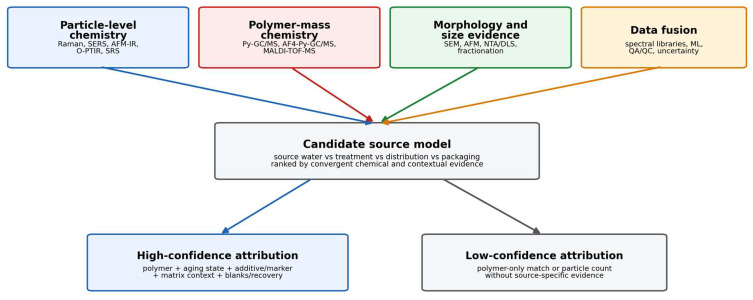
Molecular fingerprinting toolbox for drinking-water nanoplastic source attribution. Particle-level chemistry, polymer-mass chemistry, morphology, and size evidence, and data fusion should be interpreted jointly to rank candidate sources. High-confidence attribution requires convergent evidence and transparent uncertainty.

Figure 3 presents a toolbox for attribution. Particle-level chemistry, polymer-mass chemistry, morphology, size information, and data fusion each provide distinct types of evidence. High-confidence attribution requires agreement across several evidence classes. Low-confidence attribution results when polymer identity is asserted without source-compatible markers or contextual controls.

Table 4 operationalizes this logic as an evidence hierarchy for source-attribution claims. It separates simple detection from polymer identification, source-compatible evidence, convergent chemical evidence, process-resolved evidence, and validated attribution models. The revised levels include operational criteria and example claim strengths so that source statements can be written as confidence-ranked inferences rather than deterministic conclusions.

### QA/QC as the Gatekeeper of Source-Attribution Confidence

QA/QC is not a supporting detail in source attribution; it is the gatekeeper that determines whether a source claim is interpretable. At a minimum, source-attribution studies should report field blanks, procedural blanks, transport blanks when applicable, container materials, recovery experiments, matrix-matched controls, and whether laboratory background particles overlap with the target polymers. A polymer match that is also found in blanks should be treated as low-confidence unless a clear spatial, temporal, or process gradient supports the sample-specific interpretation.

Recovery is equally important because nanoplastics can adsorb to glassware, filters, tubing, and membranes. Without recovery information, a low concentration may reflect poor capture rather than low environmental burden, and an apparent source gradient may reflect workflow loss rather than true origin. QA/QC criteria should therefore be reported alongside source-confidence levels rather than placed only in methods.

## 6. Packaging-Derived Nanoplastics

Packaging-derived nanoplastics are among the most important yet difficult targets for source attribution in potable water research. Bottled water is exposure-relevant because the consumer receives the final product after it has undergone source treatment, packaging, transport, storage, and opening. Particles can therefore be introduced after conventional water-quality control. Bottle walls, caps, liners, labels, closures, and filtration infrastructure may all contribute to the observed nanoscale fraction [7,8,9,15,16].

PET attribution is attractive but not trivial. SERS detection of PET nanoplastics in bottled water supports packaging relevance, while SRS imaging has shown that particle populations in bottled water can be far larger at the nanoscale than estimates based only on micrometer-scale methods [7,8]. However, a PET signal alone does not prove that particles were shed from the bottle wall during storage. PET may also enter through source water, filtration media, procedural background, or handling. Source attribution requires packaging controls, storage experiments, cap-opening controls, matched bottle-material spectra, and, ideally, additive or oligomer profiles.

Caps and closures deserve particular attention. Microplastic studies comparing water bottled in PET, recycled PET, and glass have shown that packaging components other than the bottle body may contribute particles, and that PE caps or closure systems may contain additives [15]. For nanoplastics, the same logic should be extended to submicron fractions. Closure abrasion, torque during opening, cap-liner contact, paint or coating fragments, and repeated handling can introduce particles that are chemically distinct from the bottle wall. A clean glass bottle is not necessarily a plastic-free system if it has a polymeric cap liner or painted closure.

The ideal packaging-attribution experiment would compare source water, water immediately after bottling, water after controlled storage, water after controlled opening, bottle-rinse blanks, cap-rinse blanks, and laboratory blanks. It would use both particle-level chemistry and polymer-specific mass. Without this design, packaging attribution should be written as a plausible source hypothesis rather than a confirmed origin.

## 7. Treatment-Induced Nanoplastic Transformation

Drinking-water treatment systems are not passive filters. They are dynamic chemical and physical environments that can remove particles, transform surfaces, redistribute size fractions, and potentially introduce polymeric material from process components. Coagulation, sedimentation, ozonation, chlorination, UV, filtration, activated carbon, membranes, and distribution interfaces can all change the particle population that later reaches the consumer [10,13,14,17,18,19].

A treatment plant should therefore be studied as a sequence of transformation environments. Sampling only final treated water can reveal exposure relevance but not source or process history. To attribute particles to treatment-induced transformation, data are needed across raw water, post-coagulation, post-ozonation, filtration, storage, distribution, and tap or bottled endpoints. AFM-IR combined with Py-GC/MS has already shown the value of linking particle-level evidence with polymer-specific mass in a treatment-train context [10].

Oxidation processes can be source-informative but also source-confounding. Ozone or UV/chlorine exposure may create oxygenated surfaces consistent with treatment exposure, but similar functional groups can also form through environmental photo-oxidation or storage-related aging. Chlorination may increase the release or transformation of source-relevant organic species from aged polymers, but the magnitude of transformation depends on polymer type, previous aging, disinfectant dose, pH, contact time, and matrix chemistry [17,18,19]. Therefore, treatment signatures should be interpreted conditionally and supported by before/after sampling rather than presented as universal diagnostic markers.

For monitoring, the treatment question should not be limited to removal efficiency. A unit operation may reduce total particle counts while enriching smaller fractions or altering detectability. Conversely, a process may reduce polymer mass but generate surface chemistry that enhances colloid interaction or adsorption of other compounds. Source attribution and risk interpretation will remain incomplete unless treatment studies report polymer identity, size-resolved fractions, surface chemistry, mass endpoints, and QA/QC together.

## 8. Regulatory and Monitoring Implications

Regulatory monitoring cannot be built on detection alone. A defensible monitoring program must be able to distinguish source categories, identify which stages are controllable, and determine whether observed changes reflect true environmental shifts or workflow-dependent measurement variation. Official assessments have repeatedly emphasized that analytical inconsistencies and insufficient exposure data limit the robust interpretation of health risks associated with small plastic particles [1,2,5,6]. Source attribution is therefore not an academic luxury; it is required for actionable monitoring.

For drinking-water authorities, the most relevant output may not be a single number. Particle counts, size distributions, polymer-specific mass, particle-level chemical identity, source confidence scores, and QA/QC flags answer different questions. Particle counts may support exposure screening; polymer-specific mass may support trend analysis; source confidence may guide interventions; and aging or additive profiles may indicate treatment or packaging pathways. These endpoints should be reported as complementary descriptors rather than collapsed into a single apparently precise concentration [4,22,24].

A tiered system is the most practical near-term model. Routine laboratories could screen for particle burden and polymer mass, while reference laboratories provide confirmatory source-attribution analyses using particle-level spectroscopy, thermal methods, size-resolved separation, and data fusion. The same logic is common in trace analytical chemistry: screening is broad, confirmation is specific, and source apportionment requires the highest evidence threshold.

Monitoring protocols should also separate regulatory questions from research questions. A research workflow may use complex single-particle platforms to explore transformation pathways. A regulatory workflow may initially prioritize robust polymer-specific mass and controlled QA/QC. The two can coexist if they share reference materials, standardized reporting fields, and interlaboratory comparison exercises [42,43,44,45,46,47,48,49,50].

## 9. Future Research Agenda

The next generation of drinking-water nanoplastic research should move from occurrence tables to attribution experiments. Future studies should not only ask whether particles are present, but also which source hypothesis best explains the measured molecular and particulate signal. That requires experimental designs that deliberately sample along the source-to-consumer pathway and include plausible source materials as comparators.

Reference materials are the highest priority. Current pristine spherical standards are inadequate for source attribution because real particles are irregular, aged, additive-rich, and matrix-conditioned. The field needs PET, rPET, PE, PP, PS, PVC, cap-liner, coating, and membrane-derived test materials aged under realistic potable-water conditions. These materials should include defined size distributions, surface chemistry, additive profiles, and processing histories [42,43,44,45,46].

Spectral and pyrolysis libraries should evolve from polymer-class libraries to source-relevant libraries. A source-attribution library should include pristine polymers, aged polymers, packaging components, cap liners, treatment materials, procedural blanks, and matrix-conditioned particles. It should store metadata on aging conditions, matrix chemistry, particle size, morphology, sample preparation, and instrument settings. Without this metadata, library matching can become a sophisticated form of guesswork.

Interlaboratory exercises should be designed around source attribution, not only detection. A useful trial would distribute blind samples containing mixtures of source-water particles, bottle-derived fragments, cap-derived fragments, oxidized treatment-aged particles, and non-plastic colloids. Laboratories would be evaluated not only on whether they detect nanoplastics, but also on whether they assign source hypotheses with appropriate uncertainty.

Table 5 converts these priorities into an actionable research agenda. It links each gap to its relevance, a near-term action, and a long-term goal, while assigning the relevant reference groups in the table cells. The agenda emphasizes that source attribution will require realistic aged test materials, source-specific spectral and thermal libraries, controlled packaging and treatment experiments, transparent data-fusion tools, and interlaboratory validation [42,43,44,45,46,47,48,49,50].

## 10. Conclusions

The analytical field has made major progress in detecting nanoplastics in drinking-water-relevant matrices, but source attribution remains the next scientific frontier. The core challenge is that a drinking-water nanoplastic signal is not produced by a single source or detector. It is the endpoint of environmental input, treatment processes, distribution, packaging, storage, handling, and laboratory workflow.

Molecular fingerprinting provides the bridge between detection and attribution. Polymer identity, particle morphology, aging state, additive and oligomer profiles, thermal marker ions, size distributions, and matrix context can collectively support source hypotheses. None of these dimensions is sufficient alone. PET/rPET, PE, PP, PS, PVC, PA, polyester fibers, pigmented polymers, and biofilm-coated particles become source-informative only when their interpretation is supported by controls, gradients, recovery, and independent chemical evidence.

The practical recommendation is therefore to report source attribution as a confidence-ranked inference rather than as an unqualified conclusion. Raman, SERS, AFM-IR, O-PTIR, SRS, Py-GC/MS, AF4-Py-GC/MS, MALDI-TOF-MS, NTA/DLS, SEM/AFM, and ML-based workflows each contribute partial evidence; robust interpretation requires that these evidence classes be integrated rather than substituted for one another. The field should move toward standardized evidence levels for attribution in the same way that it is moving toward standardized requirements for detection.

Closing this gap will require aged reference materials, source-specific libraries, controlled packaging and treatment experiments, and interlaboratory exercises that test not only whether nanoplastics can be detected, but whether their origin can be assigned with defensible uncertainty. Only then will drinking-water nanoplastic analysis progress from particle observation to actionable chemical understanding.

## Figures and Tables

**Table 1:** Potential sources of nanoplastics in drinking-water systems and expected source signatures [1,2,3,7,8,9,10,11,12,13,14,15,16].

Candidate Source	Likely Polymer or Material Signal	Source-Informative Evidence	Main Ambiguity
Source water [1,2,3]	Mixed PE, PP, PET, PS, PVC; weathered fragments; biofilm-coated particles	Upstream detection, temporal correlation with runoff or wastewater influence, oxidized surfaces, mixed polymer spectrum	The same polymers may also appear from packaging, distribution, or laboratory background
Treatment materials and unit operations [10,13,14,17,18,19]	PE/PVC/PP fragments, membrane-associated polymers, oxidized particles	Before/after treatment-train gradients, unit-operation mass balance, changes in surface functionality	Removal and generation may occur simultaneously
Distribution infrastructure [10,13,14,23]	Pipe, gasket, coating, deposit- or biofilm-associated polymeric fragments	Spatial increase after treatment, pipe-material compatibility, repeated household or network sampling	Infrastructure metadata is often incomplete
Bottle body [7,8,9,15,16]	PET or rPET particles, oligomers, acetaldehyde-related or packaging-compatible markers	Polymer match to bottle material, storage/handling dependence, low source-water signal	PET can also be introduced by filtration or environmental contamination
Caps, liners, labels, closures [15,16]	PE, PP, PET, polyester coatings, pigments, or paint-related fragments	Cap-material match, opening/closing abrasion, colored particles, or additive compatibility	Particles may be transferred during manufacturing or sample handling
Laboratory background [4,22,23,24]	Airborne fibers, procedural blank polymers, sampling container residues	Presence in blanks, batch-specific contamination pattern, absence of matrix gradient	Can mimic a true environmental signal at low particle burden

**Table 2:** Treatment and transformation processes relevant to source attribution [10,13,14,17,18,19,20,21,22].

Process	Expected Transformation	Attribution Value	Analytical Caution
Ozonation [18]	Surface oxidation; carbonyl/carboxyl formation; potential fragmentation or altered hydrophilicity	May indicate exposure to oxidative treatment or transformation hotspots	Oxidation can also occur environmentally, so ozone attribution requires treatment-stage sampling
Chlorination [17,18]	Polymer-specific reactions; additive/oligomer release; possible DBP precursor behavior	Can help interpret post-disinfection changes and by-product relevance	Reaction extent depends on polymer type, aging state, chlorine dose, pH, and matrix
UV and UV-based AOPs [17,19,21]	Photo-oxidation, embrittlement, radical-driven surface modification	Useful for distinguishing pre-aged vs. freshly generated particles	Laboratory accelerated aging may not reproduce real potable-water conditions
Coagulation/flocculation [10,13,14]	Selective aggregation and removal depending on charge and colloidal stability	Before/after mass balance can identify removal or breakthrough	Coagulant residues and natural colloids may interfere with nanoscale measurements
Granular filtration and biofilm-coated media [10,13,14,23]	Physical retention, biofilm interaction, and selective breakthrough	Retention patterns can support treatment-stage attribution	Biofilm-coated particles may mask polymer signatures
Membrane filtration [10,13,14]	Retention, abrasion, and possible polymeric shedding from components	Can separate source particles from process-derived particles if controls are included	Membrane materials can become a source of contamination or a vector
Storage and handling [7,8,9,15,16]	Mechanical stress, temperature effects, cap or bottle abrasion, and additive migration	Crucial for packaging attribution	Consumer and laboratory handling histories are often undocumented

**Table 3:** Molecular and analytical fingerprints for major polymers relevant to drinking-water systems [4,7,10,12,15,16,22,23,24,25,26,27,28,29,30,31,32,33,34,35].

Polymer/Material Class	Potential Drinking-Water Source	Useful Fingerprint Dimensions	Attribution Limitation
PET/rPET (recycled PET) [7,8,9,15,16]	Bottle walls, packaging, fibers, filtration materials	Raman/SERS PET bands, PET pyrolysis markers, oligomers, bottle-compatible morphology	Base PET identity alone cannot locate the release stage
PE [10,13,15]	Caps, liners, pipes, treatment components, and environmental fragments	Aliphatic spectral features, thermal markers, additive profiles, and cap compatibility	Common environmental polymer with many possible sources
PP [15,16]	Caps, closures, packaging, filtration housings, and environmental fragments	Raman/IR aliphatic signals, pyrolysis products, pigment/additive evidence	Can be confused with packaging or procedural background without blanks
PS [18,25,26,27,28,32]	Model nanoplastics, laboratory materials, and environmental inputs	Aromatic spectral features, styrene pyrolysis markers, and SERS sensitivity	Often used as a model material, real drinking-water relevance must be justified
PVC [10,13]	Pipes, fittings, treatment infrastructure, and environmental fragments	C-Cl related IR features, dehydrochlorination/thermal markers, additives	Additives and weathering can dominate chemical interpretation
Polyamide (PA)/ polyester fibers [4,22]	Textiles, airborne contamination, filtration, or packaging residues	Fiber morphology, amide/ester bands, blank co-occurrence	High risk of airborne laboratory contamination
Pigmented polymers, paints, coatings [15,16,32]	Caps, labels, internal coatings, colored closures, painted components, and industrial or procedural contamination	Color, pigment signal, coating morphology, additive package, and compatibility with labeled or coated source materials	May be misclassified as a base polymer if pigment interference is ignored
Aged or biofilm-coated particles [17,18,19,20,21,22,23,35]	Source water, treatment media, and distribution deposits	Carbonyl index, surface roughness, biofilm-associated signal, and altered hydrophobicity	An aging state may reflect multiple environments rather than one source

**Table 4:** Evidence levels for nanoplastic source-attribution claims [4,22,23,24,33,34,35,36,37,38,39,40,41,42,43,44,45,46,47,48,49,50].

Evidence Level	Operational Criteria and Minimum Evidence	Example Claim Strength	Editorial Interpretation
Level 0: detection only	Particle count, hydrodynamic size (NTA/DLS), or non-specific nanoscale signal; no polymer identity.	Nanoscale particles are present	Insufficient for nanoplastic source attribution
Level 1: polymer identification	Raman/SERS/IR or Py-GC/MS identifies polymer class; blank context is reported.	PET-like or PE-like particles are present	Useful occurrence evidence, weak source evidence
Level 2: source-compatible polymer evidence	Polymer class is compatible with bottle, cap, pipe, membrane, or source-water materials.	Packaging or treatment source is plausible	Still inferential without independent support
Level 3: convergent chemical evidence	Polymer identity plus at least one source-relevant feature: aging state, additive/oligomer marker, pyrolysis-marker pattern, or morphology.	Packaging-, treatment-, or distribution-related source is likely	Appropriate for cautious source hypothesis
Level 4: process-resolved evidence	Before/after or spatial gradient; matched source material; blanks and recovery; repeated sampling.	The specific source pathway is strongly supported	High-confidence source attribution
Level 5: validated attribution model	Interlaboratory-tested workflow using reference materials, spectral libraries, uncertainty model, and source controls.	Attribution is operationally defensible for monitoring	Regulatory-grade evidence

**Table 5:** Priority research gaps for source attribution of nanoplastics in drinking-water systems [42,43,44,45,46,47,48,49,50].

Research Gap	Why it Matters	Near-Term Action	Long-Term Goal
Aged reference materials [42,43,44,45,46]	Pristine spheres do not represent real source particles	Produce bottle-, cap-, pipe-, membrane-, and treatment-aged test materials	Certified or consensus reference materials for attribution workflows
Source-specific libraries [32,33,34,36,37,38,39,42,43,44,45,46,47,48,49,50]	Polymer-class matching is not enough for origin assignment	Build Raman/SERS/IR/Py-GC/MS libraries with source metadata	Shared open libraries with uncertainty and QA/QC descriptors
Packaging experiments [7,8,9,15,16]	Bottled-water particles may arise from multiple packaging components	Controlled storage, opening, cap-rinse, bottle-rinse, and source-water controls	Component-resolved packaging attribution
Treatment-train experiments [10,13,14,17,18,19]	Treatment can remove, transform, or generate source signatures	Before/after sampling across unit operations with matched endpoints	Process-resolved transformation models
Data fusion and chemometrics [36,37,38,47,48,49,50]	Source attribution requires multiple evidence classes	Develop transparent scoring systems and validation datasets	Interoperable attribution models with confidence categories
Interlaboratory trials [42,43,44,45,46,47,48,49,50]	Source claims must survive method transfer	Blind samples with known mixed sources and matrix interferences	Regulatory-grade attribution protocols

## Data Availability

No new data were created or analyzed in this study. Data sharing is not applicable to this article.

## Abbreviations

AF4, asymmetric flow field-flow fractionation; AFM, atomic force microscopy; AFM-IR, atomic force microscopy–infrared spectroscopy; DBP, disinfection by-product; DLS, dynamic light scattering; FTIR, Fourier-transform infrared spectroscopy; MALDI-TOF-MS, matrix-assisted laser desorption/ionization time-of-flight mass spectrometry; ML, machine learning; NTA, nanoparticle tracking analysis; O-PTIR, optical photothermal infrared spectroscopy; PA, polyamide; PE, polyethylene; PET, poly(ethylene terephthalate); PP, polypropylene; PS, polystyrene; PVC, poly(vinyl chloride); Py-GC/MS, pyrolysis–gas chromatography–mass spectrometry; QA/QC, quality assurance/quality control; rPET, recycled poly(ethylene terephthalate); SEM, scanning electron microscopy; SERS, surface-enhanced Raman spectroscopy; SRS, stimulated Raman scattering microscopy.
